# Oligonucleotide-Based Therapies for Chronic HBV Infection: A Primer on Biochemistry, Mechanisms and Antiviral Effects

**DOI:** 10.3390/v14092052

**Published:** 2022-09-16

**Authors:** Andrew Vaillant

**Affiliations:** Replicor Inc., 6100 Royalmount Avenue, Montreal, QC H4P 2R2, Canada; availlant@replicor.com

**Keywords:** NAP, ASO, siRNA, HBsAg, functional cure, immunostimulation

## Abstract

Three types of oligonucleotide-based medicines are under clinical development for the treatment of chronic HBV infection. Antisense oligonucleotides (ASOs) and synthetic interfering RNA (siRNA) are designed to degrade HBV mRNA, and nucleic acid polymers (NAPs) stop the assembly and secretion of HBV subviral particles. Extensive clinical development of ASOs and siRNA for a variety of liver diseases has established a solid understanding of their pharmacodynamics, accumulation in different tissue types in the liver, pharmacological effects, off-target effects and how chemical modifications and delivery approaches affect these parameters. These effects are highly conserved for all ASO and siRNA used in human studies to date. The clinical assessment of several ASO and siRNA compounds in chronic HBV infection in recent years is complicated by the different delivery approaches used. Moreover, these assessments have not considered the large clinical database of ASO/siRNA function in other liver diseases and known off target effects in other viral infections. The goal of this review is to summarize the current understanding of ASO/siRNA/NAP pharmacology and integrate these concepts into current clinical results for these compounds in the treatment of chronic HBV infection.

## 1. Introduction

**Figure 1:**
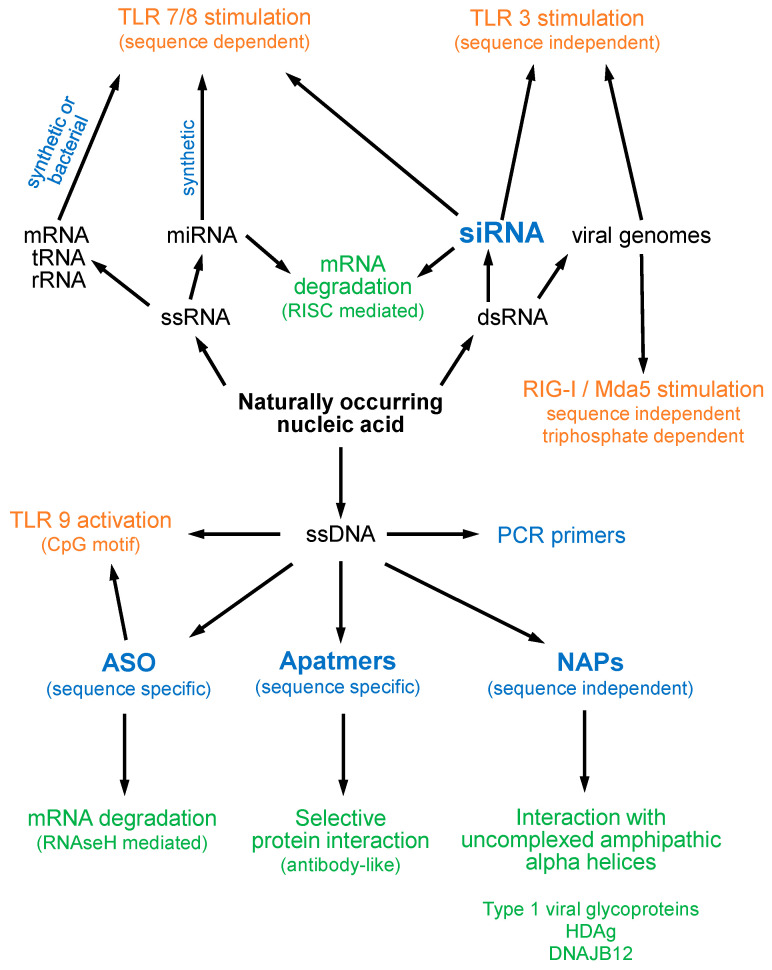
Relationships between known oligonucleotide classes and their pharmacological activities. Synthetic oligonucleotides are indicated in blue with therapeutic activity in bold. Non-immune pharmacological effects are indicated in green. Immunostimulatory effects are indicated in orange.

There are currently four known types of oligonucleotide therapeutic functionality (Figure 1). These include directing cleavage of mRNA, stimulation of innate immunity, sequence specific protein interaction (aptamers) and sequence-independent protein interactions (nucleic acid polymers or NAPs). The most well-known oligonucleotide activity is the sequence specific cleavage of mRNA by antisense oligonucleotides (ASOs) and synthetic interfering double stranded RNA (siRNA). These have been extensively studied for more than two decades, with several ASO- and siRNA-based drugs now approved for the treatment of several different liver-related diseases [1]. ASOs direct mRNA cleavage via their sequence-specific hybridization to a complimentary mRNA sequence. The resulting ASO/mRNA (DNA/RNA) hybrid recruits RNAseH and results in rapid site-specific mRNA cleavage and degradation with concomitant rapid declines in target protein [1]. The initial stages of siRNA-mediated mRNA cleavage are similar to endogenous miRNA function and involve processing via Dicer and loading onto the RNA-induced silencing complex (RISC), which is then directed to a specific mRNA target sequence via complementary hybridization. An RNAseH domain in Ago2 (a RISC component) then cleaves the mRNA [1]. Both ASO and siRNA approaches are exquisitely sensitive to base pair mismatches between the ASO/siRNA and target mRNA: single base mismatches effectively prevent mRNA cleavage directed by either ASO or siRNA [2].

All oligonucleotides have the potential to stimulate the innate immune response (Figure 1). These immunostimulatory features are derived from the ability of oligonucleotides to mimic genomic features of bacteria and viruses which are recognized by pattern recognition receptors (PRRs) including Toll-like receptors (TLRs) and retinoic-acid-inducible gene 1 (RIG-I) [3]. Sequence independent PRR recognition occurs with double stranded RNA (siRNA) via TLR3 or RIG-I [4]. Sequence dependent PRR recognition occurs with the presence of CpG motifs in DNA via TLR9 [5] or with single or double stranded RNA (siRNA) [6,7] via TLR7 [8] and TLR8 [9].

Interactions between oligonucleotides and proteins are driven by sequence dependent and sequence independent mechanisms (Figure 1). Sequence specific adoption of complex secondary and tertiary structure by oligonucleotides (aptamers) can interact with specific proteins in an antibody-like fashion [10]. TLR recognition is an example of an aptameric interaction but these are also engineered with specific oligonucleotide sequences for therapeutic, diagnostic or industrial applications [10]. Sequence independent protein interactions have been well studied for ASOs and include a conserved but diverse range of serum proteins which influence ASO pharmacokinetics and tissue distribution [11]. Nucleic acid polymers (NAPs) utilize the features of long (40 mer) phosphorothioate oligonucleotides to drive high affinity interactions with uncomplexed amphipathic alpha helices [12,13] which do not occur with ASO. To date NAP applications have been driven by demonstrated interactions with type 1 viral fusion glycoproteins [14,15], prion proteins [16], hepatitis delta antigen [17] and the heat shock protein 40 kD (HSP40) chaperone J-protein DNAJB12 [18].

## 2. Oligonucleotide Modifications and Effects on Structure and Function

**Figure 2:**
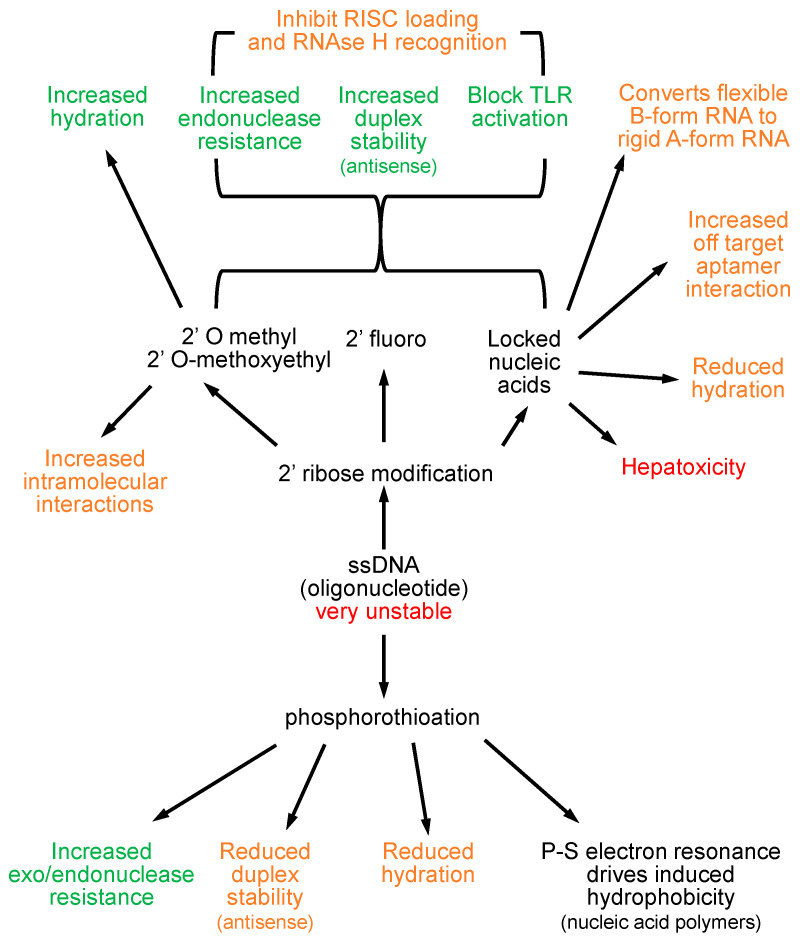
Impact of oligonucleotide modification on pharmacological effects. Positive effects are indicated in green, modifications with the potential to alter effects are indicated in orange. Negative effects are indicated in red.

Oligonucleotides are inherently unstable (Figure 2), and their therapeutic utility as ASOs could not be realized until the discovery of the phosphorothioate modification (PS) [19] as a stabilizing modification, in which one of the non-bridging oxygen atoms in the phosphodiester linkage is replaced by sulfur. PS stabilizes oligonucleotides from exo- and endonuclease attack [20], giving these compounds the stability required for the development of the first oligonucleotide therapeutics. PS also allows electron resonance between the phosphosphorous and sulfur atoms, which permits the induction of a hydrophobic moment along the oligonucleotide in the presence of a hydrophobic substrate [21], driving liver accumulation of ASOs and NAPs and forming the biochemical basis for the activity of NAPs [13]. PS modification also reduces hydration (solubility) [22] and introduces chirality in the phosphodiester linkage, lowering hybridization stability [23], which led to the implementation of a variety of ribose modifications at the 2′ position of the ribose sugar which improve duplex stability. In these second generation ASOs, 2′modified ribose modifications are used in a “gapmer” configuration with the external 4–5 ribose sugars being 2′ modified on the 5′ and 3′ ends of the oligonucleotide, leaving a central DNA core capable of eliciting RNAseH activity (see below).

**Figure 3:**
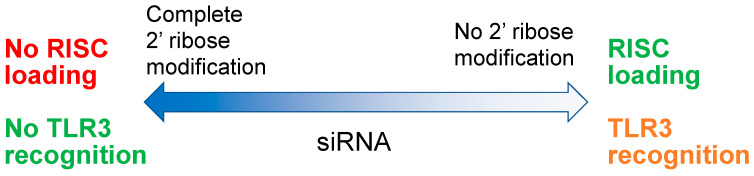
The balance between siRNA modification RISC loading and TLR3 recognition.

O-linked methylation at the 2′ position of ribose (2′OMe) is a naturally occurring nucleotide modification in eukaryotes, which is rarely found in bacterial and viral RNAs. The natural inhibition of PRR recognition by all oligonucleotides by 2′OMe modification [24] was the foundation for the introduction of 2′ ribose modifications to reduce the proinflammatory activities of early ASOs (second generation ASOs) and siRNA [25]. Additionally, 2′ribose modification also improves ASO nuclease stability, ASO/mRNA duplex stability and hydration of oligos [26,27,28], preventing off target protein interactions and improving the therapeutic index of ASOs. Ribose modification at the 2′ position of oligonucleotides now routinely includes 2′OMe, 2′ methoxyethyl (2′MOE), 2′ fluoro or locked nucleic acids (LNA, 2′-4′ ethylene bridging of ribose) [29,30]. There are caveats to the use of these modifications; 2′ ribose modification also blocks RNAseH recognition of ASO/mRNA duplexes so retention of the ASO effect requires a “gapmer” configuration of 2′ ribose modification [31], leaving at least 8 DNA nucleotides in the center of the ASO which have 2′H ribose [32,33]. The increased hybridization stability from 2′ ribose modification can also lead to the formation of a secondary structure [34], which in fully 2′ ribose modified oligonucleotides can lead to increased off-target effects. Importantly, the use of 2′ ribose modification to block immunostimulatory effects with siRNA also impacts its loading into the RISC complex (Figure 3); thus, complete removal of TLR reactive properties of siRNA are not possible without blocking RISC loading and target mRNA cleavage [35].

LNA reduces oligonucleotide hydration [36,37] and alters the structure of oligonucleotides to a rigid A-form configuration [38,39], which can interfere with oligonucleotide function [40] and drives the formation of highly stable secondary structures with various homopolymeric sequences [41,42,43]. These structural alterations direct significant off-target RNAseH mediated mRNA cleavage with LNA-modified ASOs, which results in increased hepatoxicity [44,45].

## 3. Liver Immunoreactivity, Cellular Uptake of Oligonucleotides, Lnp and Galnac

**Figure 4:**
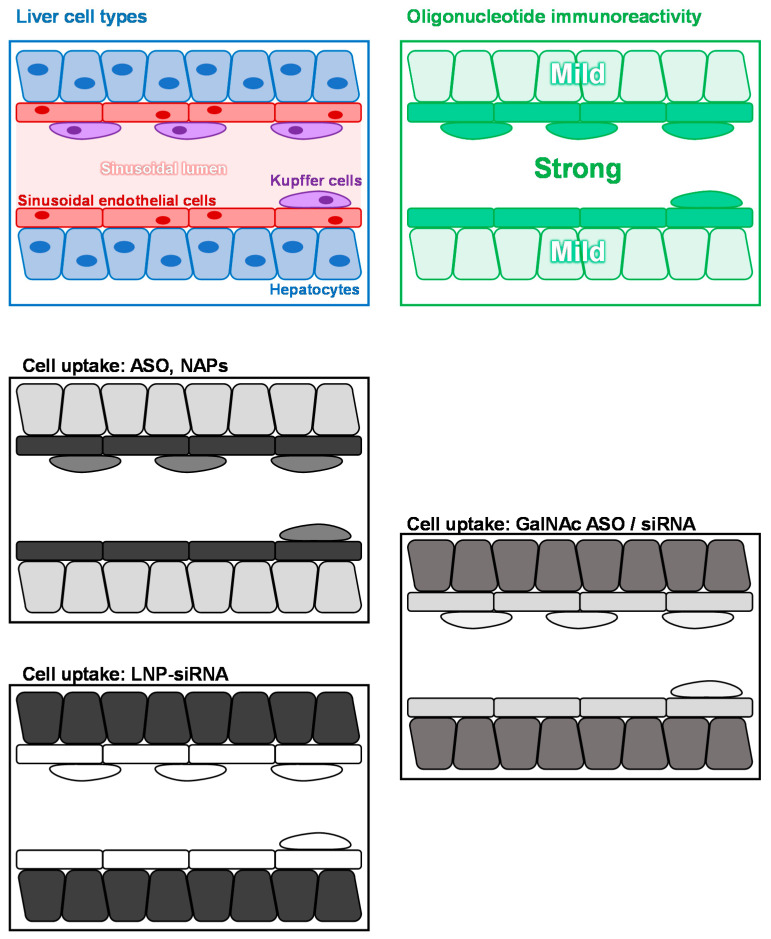
Impact of oligonucleotide uptake in the liver versus immunostimulatory impact. A map of the most prevalent liver cell types is at upper left. Relative immunoreactivity to oligonucleotides via TLR3/7/8 and 9 is indicated at upper right. Cell uptake with oligonucleotides are indicated as unconjugated ASOs (middle left), LNP formulation siRNA (lower left) and GalNAc conjugated-ASO or siRNA (lower right). Stronger cellular accumulation is indicated by darker shading.

Innate immunoreactivity driven by PRR within the liver occurs in three main cell types: hepatocytes, liver sinusoidal endothelial cells (LSECs) and Kupffer cells (KCs) (Figure 4). Within these cell types, oligonucleotide immune reactivity (TLR3/7/8/9) is most predominant in LSECs and KCs (Figure 4) [46,47,48]. PS modification of ASOs and NAPs drives their accumulation primarily in the liver and kidney [49,50,51,52] and is not affected by 2′ ribose modification. Within the liver, the majority of ASO/NAP accumulation occurs in LSECs, with smaller accumulations in KC and hepatocytes (Figure 4) [53]. The PS modification is only very sparingly used in siRNA and as such, even when stabilized with a variety of 2′ ribose modifications, very little accumulation occurs in the liver. First generation siRNA drugs used lipid nanoparticle (LNP) formulation. LNP-siRNA complexes with apolipoprotein E (Apo E) following administration and transits LSECs and KCs silently to deliver naked siRNA with high specificity and selectivity into hepatocytes (Figure 4) [54,55,56]. Unfortunately, LNPs also have proinflammatory effects in the liver [57], ultimately resulting in their discontinuation in clinical development for more recent siRNA drugs. Modern ASO and siRNA drugs in development now employ conjugation with a triennial N-acetylglucosamine moiety (GalNAc) to achieve delivery to hepatocytes [58]. This conjugation approach significantly improves the fraction of ASOs accumulating in hepatocytes and circumvents the proinflammatory effects of LNPs. However, a significant minority (~30%) of GalNAc-conjugated ASOs and siRNAs are delivered to LSEC and KC [59,60]. Thus, GalNAc conjugation has the effect of reducing the relative immunostimulatory effects of any given ASO versus its unconjugated counterpart but increases the immunostimulatory effect of any particular siRNA versus its LNP-formulated counterpart (Figure 4).

## 4. Pharmacodynamics of the ASO/siRNA Response

With the advent of GalNAc conjugation, numerous conjugated ASOs and siRNA have either undergone clinical development or have been approved for use for treatment of a variety of liver or liver-related diseases [58]. The rapid degradation of mRNA following the first dose of GalNAc-ASO or GalNAc siRNA is universally conserved both between subjects and for different target mRNAs and similar between ASO and siRNA action, resulting in a 90% (~1 log_10_) reduction in target protein production within two weeks [61,62,63,64,65,66,67,68,69,70,71]. The efficient targeting of siRNA to hepatocytes with LNP formulation allows a lower siRNA dose (~10 mg) to saturate mRNA cleavage [61,62]. As GalNAc conjugation results in a smaller fraction of drug delivered to hepatocytes vs. LNP, GalNAc conjugation of ASO and siRNA requires dosing in the 40–100 mg range to saturate mRNA cleavage [63,65,66,67,68,69,70,71].

Due to different rates of clearance from the cytoplasm, GalNAc-ASOs typically require once weekly dosing, while GalNAc-siRNA dosing can be done every 4 weeks. Suppression of protein expression beyond the 90% reduction from baseline is never observed with any ASO or siRNA, even with repeated dosing because of exhaustion/saturation of the pharmacological effect of these compounds. ASOs are constantly consumed in the RNAse H-mediated cleavage of target mRNA, RISC loading by siRNA is competed by endogenous RISC loading by miRNA [72,73] and siRNA stimulates its own degradation via activation of TLR3 [74]. However, GalNAc-conjugation has been recently shown to act as a long-term depot for siRNA in acidified compartments inside hepatocytes [75], which may explain the longer duration of siRNA availability and pharmacological effect in the liver after a single dose vs. ASOs.

## 5. Molecular Biology of HBV vs. Mechanistic Approaches of Oligonucleotide-Based Drugs

HBV infection occurs in hepatocytes and exists in two genetic forms: closed covalent circular DNA (cccDNA) minichromosomes, which are present in euchromatic (active) and heterochromatic (inactive forms) [76], and chromosomally integrated HBV DNA [77]. The copy number of cccDNA remains fairly constant over the course of chronic HBV infection [76,78] but HBV DNA integration is progressive with increasing duration of infection [77]. Active cccDNA produces virus, subviral filaments and subviral particle spheres (SVP), whereas integrated HBV DNA can only produce subviral filaments and SVP [79]. Chronic HBV infection is genetically diverse, with thousands of pre-existing quasispecies of HBV present prior to ASO/siRNA exposure with the ability to rapidly evolve in response to selection pressure from these agents [80,81]. This is due to the lack of a proofreading functionality in the HBV reverse transcriptase and the rapid turnover of transcriptionally active cccDNA [82]. Chronicity of HBV infection is maintained by the persistent and abundant circulating levels of HBsAg. HBsAg has diverse immunosuppressive properties against both innate and adaptive immunity and prevents the establishment of host immune control [79]. Targeting of HBsAg has been difficult to achieve because > 99.99% is derived from SVP, which is produced independently from viral replication and independently from cccDNA activity via integrated HBV DNA [79]. As such, currently approved direct acting antiviral therapies for chronic HBV infection, such as entecavir (ETV), tenofovir disoproxil fumarate (TDF) or tenofovir alafenamide (TAF), work well to suppress viral replication but require life-long treatment and still result in the development of HCC if not started early in infection.

The current challenge in the treatment of chronic HBV is the establishment of a functional cure which entails clearance of HBsAg during therapy to permit the restoration of immune control and normal liver function persisting in the absence of therapy. All of the agents currently in development to target HBsAg are oligonucleotide-based: a single unconjugated ASO (bepirovirsen, aka GSK3228836), several GalNAc-conjugated-siRNA (JNJ-3989 [aka ARO-HBV], VIR-2218 [aka ALN-HBV02], AB-729 and RG6346 [aka DCR-HBVS]) and NAPs (REP 2139-Mg). All ASO/siRNA consist of a single trigger targeting mRNA sequence in the HBsAg or HBx gene with the exception of JNJ-3989, which employs two triggers, one in HBsAg and one in HBx. REP 2139-Mg is the magnesium chelate complex formulation of REP 2139, which is a PS-modified 40 mer oligonucleotide with the heteropolymeric sequence (2′OMeA, 2′OMe-5MeC)_20_ [13].

## 6. Animal Models Suitable for the Evaluation of ASO, siRNA and NAPs

Several different animal models have been used to investigate the antiviral effects of oligonucleotide-based drugs for chronic HBV infection; however, important limitations and species-specific issues have been largely ignored in the analysis of in vivo data generated with ASO/siRNA in these models (Table 1). To correctly model ASO/siRNA effects in human HBV infection, pre-existing quasispecies diversity and potential for rapid evolution of escape mutants via single point mutation present in human infection are critical features. These are absent in transgenic and HDI/AAV mouse models of infection but present in duck and woodchuck models [83]. Additionally, oligonucleotide immunoreactivity in humans is poorly modeled in rodent systems [84,85]: CpG motifs which optimally simulate TLR9 differ between humans and other species [86] and TLR3 reactivity in mice is much stronger than in primates [87]. SVP production in humans is at least in part HDL based [88] and HDL metabolism is reversed in rodents relative to primates but is conserved in avian species [89]. This suggests altered mechanisms for SVP production in rodents which may explain the altered morphology of SVP in rodents vs. humans [79]. In Scid-Hu mice, the human liver explant is perfused by the host circulation but remains in a dedifferentiated state with a lack of sinusoids, the absence of bile canalicular formation between human and mouse hepatocytes and pronounced steatosis [90]. These phenotypes suggest altered lipid metabolism and oligonucleotide uptake.

## 7. NAP Effects In Vitro, In Vivo and Humans in HBV Infection

Given that the antiviral activity of NAPs is sequence independent [12], the current NAP REP 2139 was optimized to eliminate the formation of secondary structure, host genome interactions and PRR recognition [13]. This was confirmed in studies in human primary human hepatocytes [91] and in non-human primates [52]. In vitro, NAPs selectively target the assembly and secretion of SVP without affecting HBV DNA or HBV RNA production, HBeAg or HBcAg production/secretion or viral replication [92,93]. The inhibition of SVP assembly appears to target unassembled HBsAg for degradation [93]. Recently, the HSP40 chaperone DNAJB12 was shown to be required for SVP assembly and secretion and selectively targeted by NAPs [18]. Recently, the development of LNA-modified NAPs (STOPs) was attempted, but these compounds ultimately had no effect in human studies [94], primarily due to the fact that the structural rigidity imparted by LNA modification destroys NAP functionality [95]. Unfortunately, the in vitro analyses of these compounds used to support clinical studies [96] were highly flawed and shown to be artifactual [95].

**Figure 5:**
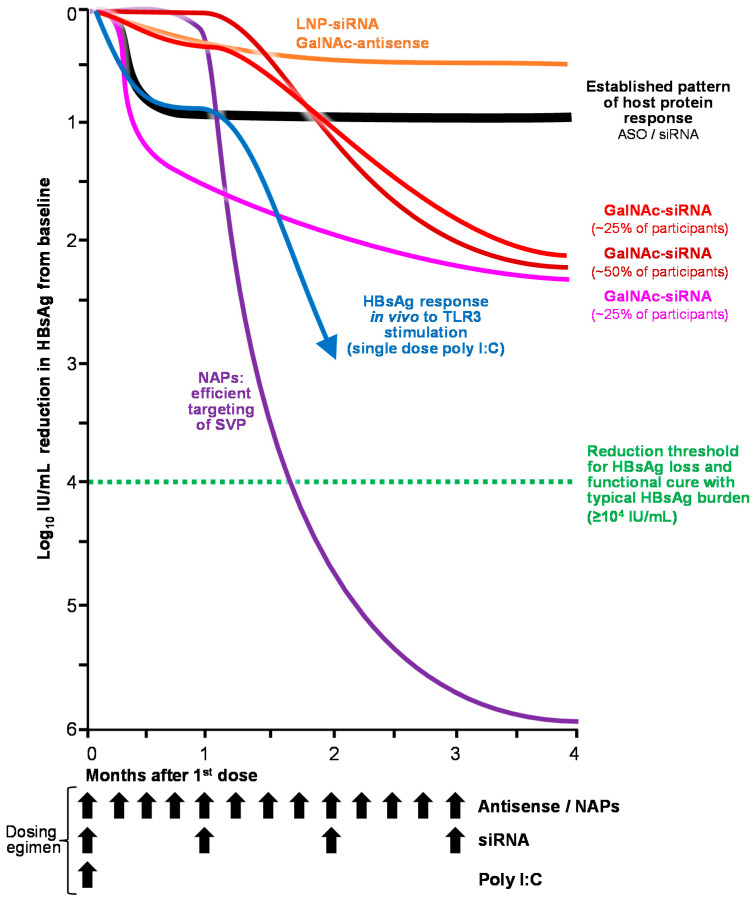
Relative impact on HBsAg response with oligonucleotide-based medicine in human chronic HBV infection.

Consistent with the issues in a variety of animal models, NAPs had no activity in transgenic mice, Scid-Hu mice or woodchuck-based HBV model systems in vivo, despite efficient liver accumulation [89]. NAP activity was easily observed in DHBV-infected ducks [97,98,99] in which selective declines in HBsAg were disconnected from HBV DNA declines (consistent with the selective targeting of SVP). Treatment with a variety of NAPs demonstrated rapid clearance of HBsAg from the circulation and liver, rapid reduction in cccDNA levels and activity in the liver and persistent immune control after removal of NAP therapy [52,97,100]. In humans, NAP monotherapy was accompanied by rapid declines in HBsAg up to 7 log_10_ from baseline (Figure 5) and by HBsAg loss and seroconversion, HBeAg seroconversion and HBV DNA clearance but limited rates of functional cure [101]. Addition of a brief (13-week) exposure to immunotherapy (thymosin α1 or pegylated interferon α2a [pegIFN]) increased the speed of HBsAg decline and the incidence of host mediated liver enzyme flares and marginally improved the incidence and duration of immune control after removal of therapy [101]. Most recently, 48 weeks of triple combination therapy with NAPs, pegIFN and TDF was accompanied by high rates of HBsAg loss and seroconversion (60%) and host-mediated transaminase flares (95%) during therapy and functional cure of HBV (39%) with an additional 39% of patients achieving partial cure after removal of therapy [102]. HBsAg loss during therapy was shown to be < 0.005 IU/mL (up to 8 log_10_ reduction from baseline) and accompanied by inactivation of cccDNA and clearance of integrated HBV DNA [103]. HBsAg isoform analysis showed selective clearance of the small isoform of HBsAg, confirming the selective targeting of SVP by NAPs in human infection [104]. A small proof of concept trial demonstrated that REP 2139 also had potent effects against HDV infection in co-infected patients, with simultaneous clearance of HDV RNA and HBsAg [105], which has persisted for 3.5 years in 7/11 and 4 of these 7 patients, respectively [106]. These antiviral responses continue to be observed in recent compassionate use of subcutaneously administered REP 2139-Mg in cirrhotic patients who failed to respond to pegIFN or pegIFN and bulevirtide [107].

## 8. ASO/siRNA Effects In Vitro, In Vivo and in Humans in HBV Infection

HepG2.2.15 cells harbor multiple copies of the HBV genome, thus providing a genetically invariable source of HBV RNA during viral replication. Early experiments with synthetic hairpin RNA (shRNA), siRNA and ASOs demonstrated antiviral activity in this and related in vitro systems [108,109,110] but without observable reductions in established cccDNA [111]. In other in vitro systems, the development of escape mutants with exogenously introduced HBV genomes were observed [112].

In vivo assessment of ASOs and siRNA almost entirely occurred in mouse models where strong antiviral responses were observed, including rapid multi-log reductions in HBsAg [113,114,115,116,117,118]. These multilog reductions in HBsAg are uniquely distinct from the normal pattern of protein response (saturation at 75–90% reduction from baseline) to ASO and siRNA observed with other liver mRNA targets in vivo [119,120,121,122,123]. However, siRNA analysis in woodchucks (which model the genetic plasticity present in human HBV infection) showed either no antiviral response or rapid rebound in viremia with continued treatment of siRNA triggers in HBsAg or HBx. This rebound was accompanied by the appearance of siRNA escape mutant mRNAs bearing point mutations in the siRNA recognition region [124], consistent with the genetic plasticity of HBV. Importantly, one of the first siRNA studies performed in the HDI mouse model of HBV infection observed cytokine induction consistent with TLR3 activation which occurred concomitantly with antiviral responses [125]. Poly I:C is a dsRNA widely used as a TLR3 agonist and in the HDI mouse model leads to a delayed and prolonged multilog HBsAg decline which is interferon dependent [126]. Moreover, polyI:C also has antiviral effects against HBV in ducks [127] and off target stimulation of innate immunity has been shown to contribute to the antiviral effects of siRNA in the woodchuck model [128].

The first clinical evaluation of siRNA in human HBV infection was performed using LNP-formulation of three siRNA triggers (2 in HBsAg and 1 in HBx, TKM-HBV/ARB-1467). This trigger combination was based on in vivo data showing futility with single triggers and designed to efficiently target mRNA produced from cccDNA or integrated HBV DNA within hepatocytes. In a phase II study conducted in ETV/TDF suppressed chronic HBV infection using ARB-1467 at doses above those saturating for the siRNA effect, mild initial responses in viremia (HBV RNA and HBcrAg) rebounded to baseline during dosing [129,130], consistent with the effects observed in woodchucks. HBsAg response was inconsistent with an siRNA effect: a highly variable and very small HBsAg reduction averaging ~0.25 log_10_ IU/mL from after 4 weeks followed by similarly small additional reductions following each siRNA dose. A second LNP-formulated siRNA (ARC-520) used two triggers, both in HBsAg. Phase I studies demonstrated transient increases in MCP-1 and IL-8 following dosing [131], consistent with TLR3 stimulation. In phase II studies, HBsAg response was again hypervariable and saturated with an average 0.1 log10 IU/mL reduction from baseline at 4 weeks at the lowest dose (1 mg/kg) still several times higher than required to saturate the siRNA effect with LNP formulation (0.15 mg/kg) [132]. Small but still hypervariable increases in HBsAg decline (average ~ 0.25 log_10_ IU/mL from baseline) required doses 4 times higher. Interestingly, the HBsAg response to ARC-520 was stronger in HBeAg-positive than HBeAg-negative patients, consistent with immunostimulatory effects as opposed to siRNA-mediated degradation (which should be the same regardless of HBeAg status). In HBeAg-positive patients, HBsAg declines following the first dose of ARC-520 approached 1.5 log_10_ IU/mL from baseline [132], but this was at dosing (4 mg/kg) substantially higher than required to saturate the siRNA effect. Multiple dosing of ARC-520 showed the same hypervariable and mild HBsAg response, averaging ~0.15 log10 IU/mL after 4 doses of 1 mg/kg and 0.4 log10 IU/mL after 4 doses of 2 mg/kg [133].

All siRNA drugs which followed ARC-520 transitioned away from LNP formulation to GalNAc conjugation (JNJ-3839, AB-729, VIR-2218 and RG6346), which is associated with accumulation of these siRNAs in KCs and LSECs. For all these GalANc-siRNA, single or multiple dose HBsAg responses were very similar (Figure 5) [134,135,136,137]. In the majority of patients, HBsAg response was either absent until 4 weeks following the first dose (similar to poly I:C response in mice) or was very mild and hypervariable with negligible HBsAg response observed at 15 days. After 4 weeks, a universal decline in HBsAg was observed in all patients saturating at 1.5–2.5 log_10_ IU/mL reduction from baseline. Despite delayed HBsAg declines in most patients, declines in markers of cccDNA activity (HBV DNA, HBV RNA and HBcrAg) were rapid [134], consistent with cccDNA inactivation. Extension of therapy to 48 weeks in NUC suppressed subjects with the addition a capsid assembly modulator did not significantly improve this response, and HBsAg rebound following cessation of treatment has been slow but continuous [134]. The addition of pegIFN only improves HBsAg decline seen with siRNA by an additional 0.5 log_10_ IU/mL [138]. To date, HBsAg loss has not occurred with any siRNA agent, except for 2 cases with ARC-520 with very low baseline HBsAg (<10 IU/mL) and with several years of exposure in the presence of ETV or TDF [139]. Importantly, HBsAg isoform response to siRNA treatment has been performed with AB-729 using the identical assay platform used for NAPs: no selective decline in the small isoform of HBsAg was observed, indicating that SVP are not targeted and that an siRNA effect is absent. Moreover, selective declines in the virus-specific large HBsAg isoform are observed, suggesting that cccDNA inactivation or other off-target effects are occurring. This is supported by additional recent observations that HBsAg response to siRNA (AB-729) is correlated with increased immunostimulatory activity [140,141].

HBsAg response to GalNAc-ASOs (RG6004/RO7062931 and GSK3389404) has also been inconsistent with an ASO effect [142,143]. No HBsAg response to RG6004 was observed with 0.5 mg/kg dosing, a dose level which yields easily observable protein reductions with Gal-NAc ASOs for other target mRNAs within 2 weeks after the first dose. Elevated dosing with RG6004 to 3 mg/kg produced a highly variable HBsAg decline between subjects which averaged only 0.4 log_10_ IU/mL after 8 weeks. Responses with GSK3398404 were almost identical to RG6004 in dose response, variability and average HBsAg decline. Bepirovirsen is the unconjugated variant of GSK3389404 carrying the identically modified oligonucleotide. This ASO is a 2′MOE gapmer designed to target mRNA cleavage in HBsAg but it also contains a class II CpG motif within its middle segment of DNA which is not shielded by 2′ ribose modification [79,144]. In contrast to administration of GSK3389404, where this oligonucleotide is delivered primarily to hepatocytes, administration of bepirovirsen results in the delivery of this oligonucleotide mainly to LSECs and KCs. With bepirovirsen, no HBV DNA or HBsAg response was observed in 4/6 subjects at the 150 mg dose [145], a dose which results in easily observed rapid protein reductions with unconjugated ASOs against other liver targets [146,147]. HBsAg responses were limited to the 300 mg dose, but in contrast to GSK 3389404, strong and rapid HBsAg declines (accompanied by host mediated transaminase flares) were restricted to subjects with baseline HBsAg < 1000 IU/mL [145]. In subjects with baseline HBsAg > 1000 IU/mL, HBsAg declines were indistinguishable from those observed with GSK3389404. Recently, HBsAg declines with bepirovirsen have also been correlated with its immunostimulatory activity [148]. These immunostimulatory activities have been attributed to TLR8 based on studies in mice but mice do not accurately model TLR reactivity in humans (Table 1) and this finding is at odds with the well understood biochemical properties of oligonucleotides: TLR8 reacts to unmodified ssRNA and TLR reactivity to TLR7/8 is well shielded by 2′MOE modification in bepirovirsen. Moreover, the oral TLR8 antagonist selgantolimod (GS-9688) did not elicit any HBsAg responses in human infection [149] despite clear immunostimulatory effects [149,150].

## 9. Conclusions and Perspective

HBsAg loss is an important milestone in the establishment of a functional cure for chronic hepatitis B infection. The molecular biology of HBV, and clinical experience with pegIFN and NAPs (the only agents currently capable of achieving HBsAg loss on therapy) indicate the antiviral effects on HBsAg required to achieve functional cure are 1) SVP particle production must be controlled and 2) HBsAg reduction > 4 log_10_ IU/mL from baseline must occur during therapy.

The genetic plasticity of chronic HBV infection is at odds with the notion that sequence-dependent mRNA cleavage of HBV mRNA by ASO or siRNA approaches are by themselves capable of achieving HBsAg responses below the threshold required for achieving functional cure. The failure of siRNA in preclinical development in models of HBV infection that model this genetic plasticity and the seminal siRNA assessment with ARB-1467, where rebound of viremia in the presence of three siRNA triggers occurred are consistent with these realities.

GalNAc-ASOs and GalNAc-siRNA have an identical pharmacodynamic protein response signature, susceptibility to single point mutation and share similar, very well-established threshold dosing for effects in humans. As such, clinical evaluations with ASOs in chronic HBV infection inform on the potential for siRNA effects and vice versa in human studies. GalNAc-ASOs are accompanied by little to no HBsAg response at doses saturatingthe ASO effect for other mRNA targets in the liver and at higher doses experience weak and hypervariable HBsAg responses still inconsistent with the ASO effect. Strong HBsAg responses are only observed when an ASO containing a TLR9 stimulatory motif is targeted to immunoreactive cells in the liver (bepirovirsen) and then only in patients with low baseline HBsAg (where immune functioning against HBV is more permissive). Consistent with these ASO findings in chronic HBV infection, HBsAg responses to all GalANc-siRNA are also inconsistent with the cleavage of mRNA. Rebound of infection occurs with LNP-RNA or mild HBsAg response is restricted to immunosensitive (HBeAg+) patient populations at siRNA doses far above the threshold required to saturate the siRNA effect. All GalNAc siRNAs have a delayed HBsAg response in the majority of patients which excludes an siRNA effect but is consistent with the exposure of immunoreactive siRNA in LSECs and KCs with GalNAc conjugation and with the delayed HBsAg responses observed with TLR 3 stimulation in mouse HBV models with dsRNA (poly I:C). These off target effects have been previously observed during the development of siRNA for influenza infection [151]. Additionally, siRNA has been shown to not target the production of SVP both directly by HBsAg isoform analysis and indirectly by the ubiquitously saturated HBsAg decline of 1.5–2.5 log_10_ IU/mL from baseline with all siRNA. SVP constitute > 99.99% of circulating HBsAg so the relatively mild HBsAg reduction threshold with siRNA indicates SVP are not being targeted.

The residence time of NAPs differs from GalNAc-ASO and GalNAc-siRNA. NAPs are rapidly cleared from the liver [52], whereas the GalNAc conjugation acts as a depot for ASO and siRNA in the liver [75]. Caution must be taken when interpreting the off-therapy antiviral responses to GalNAc-ASO and GalNAC-siRNA as the pharmacological effects of these agents can linger for months. As such, the delayed or slow rebound of HBsAg observed following cessation of GalNAc-ASO or GalNAc siRNA may reflect persistent pharmacological effects of these agents instead of real host antiviral responses.

Based on the substantial amount of available clinical data, the persisting or rapid development of escape mutants to ASO or siRNA exposure appear to restrict the effects of siRNA and ASO in human HBV infection to inactivation of cccDNA and or autophagy of HBsAg destined to form virus or subviral filaments: both activities are known to be stimulated by TLR activation. Neither ASO or siRNA appear to directly target the degradation of mRNA and consequently, the production of SVP from integrated HBV DNA. This is an important distinction as integrated HBV DNA is the source of the bulk of HBsAg in HBeAg negative chronic HBV infection [79]. On the other hand, NAPs do not directly affect viral replication but very effectively target SVP production from cccDNA and integrated HBV DNA, leading to much stronger HBsAg declines to below the limit of detection of the most sensitive experimental HBsAg assay available.

It is important to point out that the apparent TLR-stimulatory effects of ASO and siRNA are the first TLR-agonist effects to yield significant antiviral responses in chronic HBV infection. This is likely a function of their ability to activate TLR3 or TLR9 and be targeted directly to the liver and to immunoreactive non-parenchymal cells. These activities will be important to consider in future combination therapies in the pursuit of HBV functional cure. In the design and reporting of future clinical data from ASO/siRNA based drugs, individual baseline HBsAg and HBsAg responses will also be important to disclose to solidify our understanding of how ASO/siRNA technologies may participate in functional cure of HBV.

## Figures and Tables

**Table 1:** Suitability of animal models of HBV infection for evaluating oligonucleotide-based therapies.

Model	SVP Production	Genetic Diversity (Pre-Existing ASO/Sirna Escape Mutants)	Rapid Turnover of cccDNA (Evolution of ASO/RNAi Escape Mutants)	TLR9 Activity (CpG DNA)	TLR3 Reactivity (dsRNA/RNAi)
Human	LDL-based (SVP are spherical)	Present	Present	Present (KCs)	Present (LSECs, KCs)
Transgenic mice	LDL metabolism opposite to humans (SVP are octahedral)	None	Present but turnover unknown	Yes but human and rodent CpG sequences differ	Stronger vs. primate
AAV/HDI-mice	LDL metabolism opposite to humans (SVP are octahedral)	None	Present but turnover unknown	Yes but human and rodent CpG sequences differ	Stronger vs. primate
Scid-Hu mice	SVP production is attenuated (altered lipid metabolism)	Present (limited due to short term infection)	Yes	Yes but human and rodent CpG sequences differ	Stronger vs. primate
Ducks	LDL metabolism similar to humans	Present (limited due to short term infection)	Yes	Yes but via altered reactivity by TLR15	Similar to primate
Woodchucks	LDL metabolism opposite to humans	Present (chronic infection)	Yes	Yes but human and rodent CpG sequences differ	Similar to primate

## Data Availability

Not applicable.

## References

[1] Crooke S.T., Baker B.F., Crooke R.M., Liang X.H. (2021). Antisense technology: An overview and prospectus. Nat. Rev. Drug Discov..

[2] Vickers T.A., Koo S., Bennett C.F., Crooke S.T., Dean N.M., Baker B.F. (2003). Efficient reduction of target RNAs by small interfering RNA and RNase H-dependent antisense agents. A comparative analysis. J. Biol. Chem..

[3] Kawai T., Akira S. (2009). The roles of TLRs, RLRs and NLRs in pathogen recognition. Int. Immunol..

[4] Robbins M., Judge A., MacLachlan I. (2009). siRNA and innate immunity. Oligonucleotides.

[5] Krieg A.M. (2002). CpG motifs in bacterial DNA and their immune effects. Annu. Rev. Immunol..

[6] Judge A.D., Sood V., Shaw J.R., Fang D., McClintock K., MacLachlan I. (2005). Sequence-dependent stimulation of the mammalian innate immune response by synthetic siRNA. Nat. Biotechnol..

[7] Sioud M. (2005). Induction of inflammatory cytokines and interferon responses by double-stranded and single-stranded siRNAs is sequence-dependent and requires endosomal localization. J. Mol. Biol..

[8] Hornung V., Guenthner-Biller M., Bourquin C., Ablasser A., Schlee M., Uematsu S., Noronha A., Manoharan M., Akira S., de Fougerolles A. (2005). Sequence-specific potent induction of IFN-alpha by short interfering RNA in plasmacytoid dendritic cells through TLR7. Nat. Med..

[9] Hornung V., Barchet W., Schlee M., Hartmann G. (2008). RNA recognition via TLR7 and TLR8. Handb. Exp. Pharmacol..

[10] Dunn M.R., Jimenez R.M., Chaput J.C. (2017). Analysis of aptamer discovery and technology. Nat. Rev. Chem..

[11] Crooke S.T., Vickers T.A., Liang X.H. (2020). Phosphorothioate modified oligonucleotide-protein interactions. Nucl. Acids Res..

[12] Vaillant A. (2016). Nucleic acid polymers: Broad spectrum antiviral activity, antiviral mechanisms and optimization for the treatment of hepatitis B and hepatitis D infection. Antivir. Res..

[13] Vaillant A. (2019). REP 2139: Antiviral Mechanisms and Applications in Achieving Functional Control of HBV and HDV Infection. ACS Infect. Dis..

[14] Vaillant A., Juteau J.M., Lu H., Liu S., Lackman-Smith C., Ptak R., Jiang S. (2006). Phosphorothioate oligonucleotides inhibit human immunodeficiency virus type 1 fusion by blocking gp41 core formation. Antimicrob. Agents Chemother..

[15] Lee A.M., Rojek J.M., Gundersen A., Stroher U., Juteau J.M., Vaillant A., Kunz S. (2008). Inhibition of cellular entry of lymphocytic choriomeningitis virus by amphipathic DNA polymers. Virology.

[16] Kocisko D.A., Vaillant A., Lee K.S., Arnold K.M., Bertholet N., Race R.E., Olsen E.A., Juteau J.M., Caughey B. (2006). Potent antiscrapie activities of degenerate phosphorothioate oligonucleotides. Antimicrob. Agents Chemother..

[17] Shamur M.M., Peri-Naor R., Mayer R., Vaillant A. (2017). Interaction of nucleic acid polymers with the large and small forms of hepatitis delta antigen protein. Hepatology.

[18] Boulon R., Angelo L., Blanchet M., Vaillant A., Labonte P. (2021). PH-dependent Interaction of NAPs with the HSP40 Chaperone DnaJB12. Hepatology.

[19] Eckstein F. (1970). Nucleoside phosphorothioates. J. Am. Chem. Soc..

[20] Spitzer S., Eckstein F. (1988). Inhibition of deoxyribonucleases by phosphorothioate groups in oligodeoxyribonucleotides. Nucl. Acids Res..

[21] Eckstein F. (2000). Phosphorothioate oligodeoxynucleotides: What is their origin and what is unique about them?. Antisense Nucl. Acid Drug Dev..

[22] White A.P., Reeves K.K., Snyder E., Farrell J., Powell J.W., Mohan V., Griffey R.H. (1996). Hydration of single-stranded phosphodiester and phosphorothioate oligodeoxyribonucleotides. Nucl. Acids Res..

[23] Jaroszewski J.W., Clausen V., Cohen J.S., Dahl O. (1996). NMR investigations of duplex stability of phosphorothioate and phosphorodithioate DNA analogues modified in both strands. Nucl. Acids Res..

[24] Sioud M., Furset G., Cekaite L. (2007). Suppression of immunostimulatory siRNA-driven innate immune activation by 2’-modified RNAs. Biochem. Biophys. Res. Commun..

[25] Judge A.D., Bola G., Lee A.C., MacLachlan I. (2006). Design of noninflammatory synthetic siRNA mediating potent gene silencing in vivo. Mol. Ther..

[26] Meidan V.M., Cohen J.S., Amariglio N., Hirsch-Lerner D., Barenholz Y. (2000). Interaction of oligonucleotides with cationic lipids: The relationship between electrostatics, hydration and state of aggregation. Biochim. Biophys. Acta.

[27] Egli M., Pallan P.S. (2007). Insights from crystallographic studies into the structural and pairing properties of nucleic acid analogs and chemically modified DNA and RNA oligonucleotides. Annu. Rev. Biophys. Biomol. Struct..

[28] Wan W.B., Seth P.P. (2016). The Medicinal Chemistry of Therapeutic Oligonucleotides. J. Med. Chem..

[29] Shen W., De Hoyos C.L., Migawa M.T., Vickers T.A., Sun H., Low A., Bell T.A., Rahdar M., Mukhopadhyay S., Hart C.E. (2019). Chemical modification of PS-ASO therapeutics reduces cellular protein-binding and improves the therapeutic index. Nat. Biotechnol..

[30] Chen S., Le B.T., Chakravarthy M., Kosbar T.R., Veedu R.N. (2019). Systematic evaluation of 2’-Fluoro modified chimeric antisense oligonucleotide-mediated exon skipping in vitro. Sci. Rep..

[31] Monia B.P., Lesnik E.A., Gonzalez C., Lima W.F., McGee D., Guinosso C.J., Kawasaki A.M., Cook P.D., Freier S.M. (1993). Evaluation of 2’-modified oligonucleotides containing 2’-deoxy gaps as antisense inhibitors of gene expression. J. Biol. Chem..

[32] Yu D., Iyer R.P., Shaw D.R., Lisziewicz J., Li Y., Jiang Z., Roskey A., Agrawal S. (1996). Hybrid oligonucleotides: Synthesis, biophysical properties, stability studies, and biological activity. Bioorg. Med. Chem..

[33] McKay R.A., Miraglia L.J., Cummins L.L., Owens S.R., Sasmor H., Dean N.M. (1999). Characterization of a potent and specific class of antisense oligonucleotide inhibitor of human protein kinase C-alpha expression. J. Biol. Chem..

[34] Abou Assi H., Rangadurai A.K., Shi H., Liu B., Clay M.C., Erharter K., Kreutz C., Holley C.L., Al-Hashimi H.M. (2020). 2’-O-Methylation can increase the abundance and lifetime of alternative RNA conformational states. Nucl. Acids Res..

[35] Leuschner P.J., Ameres S.L., Kueng S., Martinez J. (2006). Cleavage of the siRNA passenger strand during RISC assembly in human cells. EMBO Rep..

[36] Kaur H., Arora A., Wengel J., Maiti S. (2006). Thermodynamic, counterion, and hydration effects for the incorporation of locked nucleic acid nucleotides into DNA duplexes. Biochemistry.

[37] Pande V., Nilsson L. (2008). Insights into structure, dynamics and hydration of locked nucleic acid (LNA) strand-based duplexes from molecular dynamics simulations. Nucl. Acids Res..

[38] Kierzek E., Pasternak A., Pasternak K., Gdaniec Z., Yildirim I., Turner D.H., Kierzek R. (2009). Contributions of stacking, preorganization, and hydrogen bonding to the thermodynamic stability of duplexes between RNA and 2’-O-methyl RNA with locked nucleic acids. Biochemistry.

[39] Bohr H.G., Shim I., Stein C., Orum H., Hansen H.F., Koch T. (2017). Electronic Structures of LNA Phosphorothioate Oligonucleotides. Mol. Ther. Nucl. Acids.

[40] Shimo T., Tachibana K., Kawawaki Y., Watahiki Y., Ishigaki T., Nakatsuji Y., Hara T., Kawakami J., Obika S. (2019). Enhancement of exon skipping activity by reduction in the secondary structure content of LNA-based splice-switching oligonucleotides. Chem. Commun..

[41] Dash C., Marino J.P., Le Grice S.F. (2006). Examining Ty3 polypurine tract structure and function by nucleoside analog interference. J. Biol. Chem..

[42] Pabon-Martinez Y.V., Xu Y., Villa A., Lundin K.E., Geny S., Nguyen C.H., Pedersen E.B., Jorgensen P.T., Wengel J., Nilsson L. (2017). LNA effects on DNA binding and conformation: From single strand to duplex and triplex structures. Sci. Rep..

[43] Xu Y., Gissberg O., Pabon-Martinez Y.V., Wengel J., Lundin K.E., Smith C.I.E., Zain R., Nilsson L., Villa A. (2019). The ability of locked nucleic acid oligonucleotides to pre-structure the double helix: A molecular simulation and binding study. PLoS ONE.

[44] Burel S.A., Hart C.E., Cauntay P., Hsiao J., Machemer T., Katz M., Watt A., Bui H.H., Younis H., Sabripour M. (2016). Hepatotoxicity of high affinity gapmer antisense oligonucleotides is mediated by RNase H1 dependent promiscuous reduction of very long pre-mRNA transcripts. Nucl. Acids Res..

[45] Kasuya T., Hori S., Watanabe A., Nakajima M., Gahara Y., Rokushima M., Yanagimoto T., Kugimiya A. (2016). Ribonuclease H1-dependent hepatotoxicity caused by locked nucleic acid-modified gapmer antisense oligonucleotides. Sci. Rep..

[46] Freitas-Lopes M.A., Mafra K., David B.A., Carvalho-Gontijo R., Menezes G.B. (2017). Differential Location and Distribution of Hepatic Immune Cells. Cells.

[47] Faure-Dupuy S., Vegna S., Aillot L., Dimier L., Esser K., Broxtermann M., Bonnin M., Bendriss-Vermare N., Rivoire M., Passot G. (2018). Characterization of Pattern Recognition Receptor Expression and Functionality in Liver Primary Cells and Derived Cell Lines. J. Innate Immun..

[48] Werner M., Schefczyk S., Trippler M., Treckmann J.W., Baba H.A., Gerken G., Schlaak J.F., Broering R. (2022). Antiviral Toll-like Receptor Signaling in Non-Parenchymal Liver Cells Is Restricted to TLR3. Viruses.

[49] Raynaud F.I., Orr R.M., Goddard P.M., Lacey H.A., Lancashire H., Judson I.R., Beck T., Bryan B., Cotter F.E. (1997). Pharmacokinetics of G3139, a phosphorothioate oligodeoxynucleotide antisense to bcl-2, after intravenous administration or continuous subcutaneous infusion to mice. J. Pharmacol. Exp. Ther..

[50] Geary R.S., Yu R.Z., Watanabe T., Henry S.P., Hardee G.E., Chappell A., Matson J., Sasmor H., Cummins L., Levin A.A. (2003). Pharmacokinetics of a tumor necrosis factor-alpha phosphorothioate 2’-O-(2-methoxyethyl) modified antisense oligonucleotide: Comparison across species. Drug Metab. Dispos..

[51] Yu R.Z., Kim T.W., Hong A., Watanabe T.A., Gaus H.J., Geary R.S. (2007). Cross-species pharmacokinetic comparison from mouse to man of a second-generation antisense oligonucleotide, ISIS 301012, targeting human apolipoprotein B-100. Drug Metab. Dispos..

[52] Roehl I., Seiffert S., Brikh C., Quinet J., Jamard C., Dorfler N., Lockridge J.A., Cova L., Vaillant A. (2017). Nucleic Acid Polymers with Accelerated Plasma and Tissue Clearance for Chronic Hepatitis B Therapy. Mol. Ther. Nucl. Acids.

[53] Bijsterbosch M.K., Manoharan M., Rump E.T., De Vrueh R.L., van Veghel R., Tivel K.L., Biessen E.A., Bennett C.F., Cook P.D., van Berkel T.J. (1997). In vivo fate of phosphorothioate antisense oligodeoxynucleotides: Predominant uptake by scavenger receptors on endothelial liver cells. Nucl. Acids Res..

[54] Akinc A., Querbes W., De S., Qin J., Frank-Kamenetsky M., Jayaprakash K.N., Jayaraman M., Rajeev K.G., Cantley W.L., Dorkin J.R. (2010). Targeted delivery of RNAi therapeutics with endogenous and exogenous ligand-based mechanisms. Mol. Ther..

[55] Shi B., Keough E., Matter A., Leander K., Young S., Carlini E., Sachs A.B., Tao W., Abrams M., Howell B. (2011). Biodistribution of small interfering RNA at the organ and cellular levels after lipid nanoparticle-mediated delivery. J. Histochem. Cytochem..

[56] Dong Y., Love K.T., Dorkin J.R., Sirirungruang S., Zhang Y., Chen D., Bogorad R.L., Yin H., Chen Y., Vegas A.J. (2014). Lipopeptide nanoparticles for potent and selective siRNA delivery in rodents and nonhuman primates. Proc. Natl. Acad. Sci. USA.

[57] Kumar V., Qin J., Jiang Y., Duncan R.G., Brigham B., Fishman S., Nair J.K., Akinc A., Barros S.A., Kasperkovitz P.V. (2014). Shielding of Lipid Nanoparticles for siRNA Delivery: Impact on Physicochemical Properties, Cytokine Induction, and Efficacy. Mol. Ther. Nucl. Acids.

[58] Debacker A.J., Voutila J., Catley M., Blakey D., Habib N. (2020). Delivery of Oligonucleotides to the Liver with GalNAc: From Research to Registered Therapeutic Drug. Mol. Ther..

[59] Prakash T.P., Graham M.J., Yu J., Carty R., Low A., Chappell A., Schmidt K., Zhao C., Aghajan M., Murray H.F. (2014). Targeted delivery of antisense oligonucleotides to hepatocytes using triantennary N-acetyl galactosamine improves potency 10-fold in mice. Nucl. Acids Res..

[60] Janas M.M., Harbison C.E., Perry V.K., Carito B., Sutherland J.E., Vaishnaw A.K., Keirstead N.D., Warner G. (2018). The Nonclinical Safety Profile of GalNAc-conjugated RNAi Therapeutics in Subacute Studies. Toxicol. Pathol..

[61] Coelho T., Adams D., Silva A., Lozeron P., Hawkins P.N., Mant T., Perez J., Chiesa J., Warrington S., Tranter E. (2013). Safety and efficacy of RNAi therapy for transthyretin amyloidosis. N. Engl. J. Med..

[62] Fitzgerald K., Frank-Kamenetsky M., Shulga-Morskaya S., Liebow A., Bettencourt B.R., Sutherland J.E., Hutabarat R.M., Clausen V.A., Karsten V., Cehelsky J. (2014). Effect of an RNA interference drug on the synthesis of proprotein convertase subtilisin/kexin type 9 (PCSK9) and the concentration of serum LDL cholesterol in healthy volunteers: A randomised, single-blind, placebo-controlled, phase 1 trial. Lancet.

[63] Graham M.J., Lee R.G., Brandt T.A., Tai L.J., Fu W., Peralta R., Yu R., Hurh E., Paz E., McEvoy B.W. (2017). Cardiovascular and Metabolic Effects of ANGPTL3 Antisense Oligonucleotides. N. Engl. J. Med..

[64] Fitzgerald K., White S., Borodovsky A., Bettencourt B.R., Strahs A., Clausen V., Wijngaard P., Horton J.D., Taubel J., Brooks A. (2017). A Highly Durable RNAi Therapeutic Inhibitor of PCSK9. N. Engl. J. Med..

[65] Liebow A., Li X., Racie T., Hettinger J., Bettencourt B.R., Najafian N., Haslett P., Fitzgerald K., Holmes R.P., Erbe D. (2017). An Investigational RNAi Therapeutic Targeting Glycolate Oxidase Reduces Oxalate Production in Models of Primary Hyperoxaluria. J. Am. Soc. Nephrol..

[66] Zimmermann T.S., Karsten V., Chan A., Chiesa J., Boyce M., Bettencourt B.R., Hutabarat R., Nochur S., Vaishnaw A., Gollob J. (2017). Clinical Proof of Concept for a Novel Hepatocyte-Targeting GalNAc-siRNA Conjugate. Mol. Ther..

[67] Sardh E., Harper P., Balwani M., Stein P., Rees D., Bissell D.M., Desnick R., Parker C., Phillips J., Bonkovsky H.L. (2019). Phase 1 Trial of an RNA Interference Therapy for Acute Intermittent Porphyria. N. Engl. J. Med..

[68] Wooddell C.I., Blomenkamp K., Peterson R.M., Subbotin V.M., Schwabe C., Hamilton J., Chu Q., Christianson D.R., Hegge J.O., Kolbe J. (2020). Development of an RNAi therapeutic for alpha-1-antitrypsin liver disease. JCI Insight.

[69] Viney N.J., Guo S., Tai L.J., Baker B.F., Aghajan M., Jung S.W., Yu R.Z., Booten S., Murray H., Machemer T. (2021). Ligand conjugated antisense oligonucleotide for the treatment of transthyretin amyloidosis: Preclinical and phase 1 data. ESC Heart Fail..

[70] McDougall R., Ramsden D., Agarwal S., Agarwal S., Aluri K., Arciprete M., Brown C., Castellanos-Rizaldos E., Charisse K., Chong S. (2022). The Nonclinical Disposition and Pharmacokinetic/Pharmacodynamic Properties of N-Acetylgalactosamine-Conjugated Small Interfering RNA Are Highly Predictable and Build Confidence in Translation to Human. Drug Metab. Dispos..

[71] Tardif J.C., Karwatowska-Prokopczuk E., Amour E.S., Ballantyne C.M., Shapiro M.D., Moriarty P.M., Baum S.J., Hurh E., Bartlett V.J., Kingsbury J. (2022). Apolipoprotein C-III reduction in subjects with moderate hypertriglyceridaemia and at high cardiovascular risk. Eur. Heart J..

[72] Bosia C., Sgro F., Conti L., Baldassi C., Brusa D., Cavallo F., Cunto F.D., Turco E., Pagnani A., Zecchina R. (2017). RNAs competing for microRNAs mutually influence their fluctuations in a highly non-linear microRNA-dependent manner in single cells. Genome Biol..

[73] Lam J.K., Chow M.Y., Zhang Y., Leung S.W. (2015). siRNA Versus miRNA as Therapeutics for Gene Silencing. Mol. Ther. Nucl. Acids.

[74] Seo G.J., Kincaid R.P., Phanaksri T., Burke J.M., Pare J.M., Cox J.E., Hsiang T.Y., Krug R.M., Sullivan C.S. (2013). Reciprocal inhibition between intracellular antiviral signaling and the RNAi machinery in mammalian cells. Cell Host Microbe.

[75] Brown C.R., Gupta S., Qin J., Racie T., He G., Lentini S., Malone R., Yu M., Matsuda S., Shulga-Morskaya S. (2020). Investigating the pharmacodynamic durability of GalNAc-siRNA conjugates. Nucl. Acids Res..

[76] Levrero M., Pollicino T., Petersen J., Belloni L., Raimondo G., Dandri M. (2009). Control of cccDNA function in hepatitis B virus infection. J. Hepatol..

[77] Tu T., Budzinska M.A., Shackel N.A., Urban S. (2017). HBV DNA Integration: Molecular Mechanisms and Clinical Implications. Viruses.

[78] Dandri M., Petersen J. (2020). cccDNA Maintenance in Chronic Hepatitis B—Targeting the Matrix of Viral Replication. Infect. Drug Resist..

[79] Vaillant A. (2020). HBsAg, Subviral Particles, and Their Clearance in Establishing a Functional Cure of Chronic Hepatitis B Virus Infection. ACS Infect. Dis..

[80] Li F., Zhang D., Li Y., Jiang D., Luo S., Du N., Chen W., Deng L., Zeng C. (2015). Whole genome characterization of hepatitis B virus quasispecies with massively parallel pyrosequencing. Clin. Microbiol. Infect..

[81] Yang Z.T., Huang S.Y., Chen L., Liu F., Cai X.H., Guo Y.F., Wang M.J., Han Y., Yu D.M., Jiang J.H. (2015). Characterization of Full-Length Genomes of Hepatitis B Virus Quasispecies in Sera of Patients at Different Phases of Infection. J. Clin. Microbiol..

[82] Huang Q., Zhou B., Cai D., Zong Y., Wu Y., Liu S., Mercier A., Guo H., Hou J., Colonno R. (2021). Rapid Turnover of Hepatitis B Virus Covalently Closed Circular DNA Indicated by Monitoring Emergence and Reversion of Signature-Mutation in Treated Chronic Hepatitis B Patients. Hepatology.

[83] Liu Y., Maya S., Ploss A. (2021). Animal Models of Hepatitis B Virus Infection-Success, Challenges, and Future Directions. Viruses.

[84] Mestas J., Hughes C.C. (2004). Of mice and not men: Differences between mouse and human immunology. J. Immunol..

[85] Zschaler J., Schlorke D., Arnhold J. (2014). Differences in innate immune response between man and mouse. Crit. Rev. Immunol..

[86] Bauer S., Kirschning C.J., Hacker H., Redecke V., Hausmann S., Akira S., Wagner H., Lipford G.B. (2001). Human TLR9 confers responsiveness to bacterial DNA via species-specific CpG motif recognition. Proc. Natl. Acad. Sci. USA.

[87] Mitchell W.M., Nicodemus C.F., Carter W.A., Horvath J.C., Strayer D.R. (2014). Discordant biological and toxicological species responses to TLR3 activation. Am. J. Pathol..

[88] Gavilanes F., Gonzalez-Ros J.M., Peterson D.L. (1982). Structure of hepatitis B surface antigen. Characterization of the lipid components and their association with the viral proteins. J. Biol. Chem..

[89] Schoneweis K., Motter N., Roppert P.L., Lu M., Wang B., Roehl I., Glebe D., Yang D., Morrey J.D., Roggendorf M. (2018). Activity of nucleic acid polymers in rodent models of HBV infection. Antivir. Res..

[90] Peterson R.A., Krull D.L., Brown H.R., de Serres M. (2010). Morphologic characterization of PhoenixBio (uPA+/+/SCID) humanized liver chimeric mouse model. Drug Metab. Lett..

[91] Real C.I., Werner M., Paul A., Gerken G., Schlaak J.F., Vaillant A., Broering R. (2017). Nucleic acid-based polymers effective against hepatitis B Virus infection in patients don’t harbor immunostimulatory properties in primary isolated liver cells. Sci. Rep..

[92] Blanchet M., Sinnathamby V., Vaillant A., Labonte P. (2019). Inhibition of HBsAg secretion by nucleic acid polymers in HepG2.2.15cells. Antivir. Res..

[93] Boulon R., Blanchet M., Lemasson M., Vaillant A., Labonte P. (2020). Characterization of the antiviral effects of REP 2139 on the HBV lifecycle in vitro. Antivir. Res..

[94] Gane E., Agarwal K., Yuen M.F., Jucov A., Schwabe C., Le K., Wang S., Westland C., Steel K., Zhang Q. (2022). Safety, pharmacokinetics, and antiviral activity of the S-antigen Transport Inhibiting Oligonucleotide Polymers (STOPS) drug candidate ALG-010133 in subjects with chronic hepatitis B. J. Hepatol..

[95] Vaillant A. (2022). Editorial: In vitro mechanistic evaluation of nucleic acid polymers: A cautionary tale. Mol. Ther. Nucl. Acids.

[96] Kao C.C., Nie Y., Ren S., Tilani N., De Costa T.S., Pamdey R.K., Hong J., Smith D.B., Symons J.A., Beigelman L. (2021). Mechanism of action of hepatitis B virus S antigen transport-inhibiting oligonucleotide polymer, STOPS, molecules. Mol. Ther. Nucl. Acids.

[97] Noordeen F., Scougall C.A., Grosse A., Qiao Q., Ajilian B.B., Reaiche-Miller G., Finnie J., Werner M., Broering R., Schlaak J.F. (2015). Therapeutic Antiviral Effect of the Nucleic Acid Polymer REP 2055 against Persistent Duck Hepatitis B Virus Infection. PLoS ONE.

[98] Noordeen F., Vaillant A., Jilbert A.R. (2013). Nucleic acid polymers inhibit duck hepatitis B virus infection in vitro. Antimicrob. Agents Chemother..

[99] Noordeen F., Vaillant A., Jilbert A.R. (2013). Nucleic acid polymers prevent the establishment of duck hepatitis B virus infection in vivo. Antimicrob. Agents Chemother..

[100] Quinet J., Jamard C., Burtin M., Lemasson M., Guerret S., Sureau C., Vaillant A., Cova L. (2018). Nucleic acid polymer REP 2139 and nucleos(T)ide analogues act synergistically against chronic hepadnaviral infection in vivo in Pekin ducks. Hepatology.

[101] Al-Mahtab M., Bazinet M., Vaillant A. (2016). Safety and Efficacy of Nucleic Acid Polymers in Monotherapy and Combined with Immunotherapy in Treatment-Naive Bangladeshi Patients with HBeAg+ Chronic Hepatitis B Infection. PLoS ONE.

[102] Bazinet M., Pantea V., Placinta G., Moscalu I., Cebotarescu V., Cojuhari L., Jimbei P., Iarovoi L., Smesnoi V., Musteata T. (2020). Safety and Efficacy of 48 Weeks REP 2139 or REP 2165, Tenofovir Disoproxil, and Pegylated Interferon Alfa-2a in Patients With Chronic HBV Infection Naive to Nucleos(t)ide Therapy. Gastroenterology.

[103] Bazinet M., Anderson M., Pantea V., Placinta G., Moscalu I., Cebotarescu V., Cojuhari L., Jimbei P., Iarovoi L., Smesnoi V. (2021). Analysis of HBsAg Immunocomplexes and cccDNA Activity During and Persisting After NAP-Based Therapy. Hepatol. Commun..

[104] Bazinet M., Anderson M., Pantea V., Placinta G., Moscalu I., Cebotarescu V., Cojuhari L., Jimbei P., Iarovoi L., Smesnoi V. (2022). HBsAg isoform dynamics during NAP-based therapy of HBeAg-negative chronic HBV and HBV/HDV infection. Hepatol. Commun..

[105] Bazinet M., Pantea V., Cebotarescu V., Cojuhari L., Jimbei P., Albrecht J., Schmid P., Le Gal F., Gordien E., Krawczyk A. (2017). Safety and efficacy of REP 2139 and pegylated interferon alfa-2a for treatment-naive patients with chronic hepatitis B virus and hepatitis D virus co-infection (REP 301 and REP 301-LTF): A non-randomised, open-label, phase 2 trial. Lancet Gastroenterol. Hepatol..

[106] Bazinet M., Pantea V., Cebotarescu V., Cojuhari L., Jimbei P., Anderson M., Gersch J., Holzmayer V., Elsner C., Krawczyk A. (2020). Persistent control of HBV and HDV infection following REP 2139-Ca and pegIFN therapy in chronic HBV/HDV co-infection. Hepatol. Commun..

[107] Bourliere M., Bazinet M., Ali S.B., Lecompte L., Vaillant A. (2021). Subcutaneous administration of REP 2139-Mg in the compassionate treatment of cirrhotic HBV/HDV co-infection. AASLD.

[108] Xin X.M., Li G.Q., Jin Y.Y., Zhuang M., Li D. (2008). Combination of small interfering RNAs mediates greater suppression on hepatitis B virus cccDNA in HepG2.2.15 cells. World J. Gastroenterol..

[109] Sun Z., Xiang W., Guo Y., Chen Z., Liu W., Lu D. (2011). Inhibition of hepatitis B virus (HBV) by LNA-mediated nuclear interference with HBV DNA transcription. Biochem. Biophys. Res. Commun..

[110] Zhang Y.L., Cheng T., Cai Y.J., Yuan Q., Liu C., Zhang T., Xia D.Z., Li R.Y., Yang L.W., Wang Y.B. (2010). RNA Interference inhibits hepatitis B virus of different genotypes in vitro and in vivo. BMC Microbiol..

[111] Starkey J.L., Chiari E.F., Isom H.C. (2009). Hepatitis B virus (HBV)-specific short hairpin RNA is capable of reducing the formation of HBV covalently closed circular (CCC) DNA but has no effect on established CCC DNA in vitro. J. Gen. Virol..

[112] Wu H.L., Huang L.R., Huang C.C., Lai H.L., Liu C.J., Huang Y.T., Hsu Y.W., Lu C.Y., Chen D.S., Chen P.J. (2005). RNA interference-mediated control of hepatitis B virus and emergence of resistant mutant. Gastroenterology.

[113] Javanbakht H., Mueller H., Walther J., Zhou X., Lopez A., Pattupara T., Blaising J., Pedersen L., Albaek N., Jackerott M. (2018). Liver-Targeted Anti-HBV Single-Stranded Oligonucleotides with Locked Nucleic Acid Potently Reduce HBV Gene Expression In Vivo. Mol. Ther. Nucl. Acids.

[114] Wooddell C.I., Rozema D.B., Hossbach M., John M., Hamilton H.L., Chu Q., Hegge J.O., Klein J.J., Wakefield D.H., Oropeza C.E. (2013). Hepatocyte-targeted RNAi therapeutics for the treatment of chronic hepatitis B virus infection. Mol. Ther..

[115] Morrissey D.V., Lockridge J.A., Shaw L., Blanchard K., Jensen K., Breen W., Hartsough K., Machemer L., Radka S., Jadhav V. (2005). Potent and persistent in vivo anti-HBV activity of chemically modified siRNAs. Nat. Biotechnol..

[116] Thi E.P., Dhillon A.P., Ardzinski A., Bidirici-Ertekin L., Cobarrubias K.D., Cuconati A., Kondratowicz A.S., Kwak K., Li A.H.L., Miller A. (2019). ARB-1740, a RNA Interference Therapeutic for Chronic Hepatitis B Infection. ACS Infect. Dis..

[117] Schlegel M.K., Janas M.M., Jiang Y., Barry J.D., Davis W., Agarwal S., Berman D., Brown C.R., Castoreno A., LeBlanc S. (2022). From bench to bedside: Improving the clinical safety of GalNAc-siRNA conjugates using seed-pairing destabilization. Nucl. Acids Res..

[118] Koser M., Craig K.P., Cyr W.A., Wang W., Brown B.D., Abrams M. (2018). Preclinical Characterization of Galxc™ RNAi Therapeutics Targeting Different Regions of the HBV Genome. Hepatology.

[119] Rajeev K.G., Nair J.K., Jayaraman M., Charisse K., Taneja N., O’Shea J., Willoughby J.L., Yucius K., Nguyen T., Shulga-Morskaya S. (2015). Hepatocyte-specific delivery of siRNAs conjugated to novel non-nucleosidic trivalent N-acetylgalactosamine elicits robust gene silencing in vivo. ChemBioChem.

[120] Cruz P.E., Mueller C., Cossette T.L., Golant A., Tang Q., Beattie S.G., Brantly M., Campbell-Thompson M., Blomenkamp K.S., Teckman J.H. (2007). In vivo post-transcriptional gene silencing of alpha-1 antitrypsin by adeno-associated virus vectors expressing siRNA. Lab. Investig..

[121] Frank-Kamenetsky M., Grefhorst A., Anderson N.N., Racie T.S., Bramlage B., Akinc A., Butler D., Charisse K., Dorkin R., Fan Y. (2008). Therapeutic RNAi targeting PCSK9 acutely lowers plasma cholesterol in rodents and LDL cholesterol in nonhuman primates. Proc. Natl. Acad. Sci. USA.

[122] Yu R.Z., Lemonidis K.M., Graham M.J., Matson J.E., Crooke R.M., Tribble D.L., Wedel M.K., Levin A.A., Geary R.S. (2009). Cross-species comparison of in vivo PK/PD relationships for second-generation antisense oligonucleotides targeting apolipoprotein B-100. Biochem. Pharmacol..

[123] Graham M.J., Lee R.G., Bell T.A., Fu W., Mullick A.E., Alexander V.J., Singleton W., Viney N., Geary R., Su J. (2013). Antisense oligonucleotide inhibition of apolipoprotein C-III reduces plasma triglycerides in rodents, nonhuman primates, and humans. Circ. Res..

[124] Lee A.M. TKM-HBV RNAi Therapeutic for Chronic HBV Infeciton. Proceedings of the DIA/FDA Oligonucleotide-Based Therapeutics Conference.

[125] Hean J., Crowther C., Ely A., Ul Islam R., Barichievy S., Bloom K., Weinberg M.S., van Otterlo W.A., de Koning C.B., Salazar F. (2010). Inhibition of hepatitis B virus replication in vivo using lipoplexes containing altritol-modified antiviral siRNAs. Artif. DNA PNA XNA.

[126] Wu J., Huang S., Zhao X., Chen M., Lin Y., Xia Y., Sun C., Yang X., Wang J., Guo Y. (2014). Poly(I:C) treatment leads to interferon-dependent clearance of hepatitis B virus in a hydrodynamic injection mouse model. J. Virol..

[127] Niu J., Wang Y., Dixon R., Bowden S., Qiao M., Einck L., Locarnini S. (1993). The use of ampligen alone and in combination with ganciclovir and coumermycin A1 for the treatment of ducks congenitally-infected with duck hepatitis B virus. Antivir. Res..

[128] Meng Z., Zhang X., Wu J., Pei R., Xu Y., Yang D., Roggendorf M., Lu M. (2013). RNAi induces innate immunity through multiple cellular signaling pathways. PLoS ONE.

[129] Agarwal K., Gane E., Cheng C., Sievert W., Roberts S.K., Ahn S.H., Kim Y.J., Streinu-Cercel A., Denning J., Symonds W. (2017). HBcrAg, HBV-RNA declines in A Phase 2a Study Evaluating the Multi-dose Activity of ARB-1467 in HBeAg-Positive and Negative Virally Suppressed Subjects with Hepatitis B. Hepatology.

[130] Eley T., Russ R., Streinu-Cercel A., Gane E.J., Roberts S.K., Ahn S.H., Kim Y.J., Symonds W., Mendez P. (2017). Pharmacokinetics and exploratory exposure-response of siRNAs administered monthly as ARB-001467 (ARB-1467) in a Phase 2a study in HBeAg positive and negative virally suppressed subjects with chronic hepatitis B. Hepatology.

[131] Schluep T., Lickliter J., Hamilton J., Lewis D.L., Lai C.L., Lau J.Y., Locarnini S.A., Gish R.G., Given B.D. (2017). Safety, Tolerability, and Pharmacokinetics of ARC-520 Injection, an RNA Interference-Based Therapeutic for the Treatment of Chronic Hepatitis B Virus Infection, in Healthy Volunteers. Clin. Pharmacol. Drug Dev..

[132] Wooddell C.I., Yuen M.F., Chan H.L., Gish R.G., Locarnini S.A., Chavez D., Ferrari C., Given B.D., Hamilton J., Kanner S.B. (2017). RNAi-based treatment of chronically infected patients and chimpanzees reveals that integrated hepatitis B virus DNA is a source of HBsAg. Sci. Transl. Med..

[133] Yuen M.F., Schiefke I., Yoon J.H., Ahn S.H., Heo J., Kim J.H., Lik Yuen Chan H., Yoon K.T., Klinker H., Manns M. (2020). RNA Interference Therapy With ARC-520 Results in Prolonged Hepatitis B Surface Antigen Response in Patients with Chronic Hepatitis B Infection. Hepatology.

[134] Yuen M.F., Locarnini S., Lim T.H., Strasser S.I., Sievert W., Cheng W., Thompson A.J., Given B.D., Schluep T., Hamilton J. (2022). Combination treatments including the small-interfering RNA JNJ-3989 induce rapid and sometimes prolonged viral responses in patients with CHB. J. Hepatol..

[135] Gane E., Lim Y.S., Cloutier D., Shen L., Cathcart A., Ding X., Pang P., Huang S., Yuen M.F. (2021). Safety and antiviral activity of VIR-2218, an X-targeting RNAi therapeutic, in participants with chronic hepatitis B infection: Week 48 follow-up results. J. Hepatol..

[136] Yuen M.F. HBV RNAi Inhibitor RG6346 in Phase 1b-2a Trial was Safe, Well Tolerated, and Resulted in Subatantial and Durable Reductions in Serum HBsAg Levels. Presented at the AASLD 2020 Late Breakong Oral Presentation.

[137] Yuen M.F., Berliba E., Sukeepaisarnjaroen W., Holmes J., Leerapun A., Tangkijvanich P., Strasser S., Jucov A., Gane E., Thi E.P. (2022). Long-term suppression maintained after cessation of AB-729 treatment and comparable on-treatment response observed in HBeAg+ subjects. J. Hepatol..

[138] Yuen M.F., Lim Y.S., Cloutier D., Shen L., Arizpe A., Pang P., Tay C., Thanawala V., Gupta S.V., Cathcart A. (2021). Preliminary on-treatment data from a phase 2 study evaluating VIR-2218 in combination with pegylated interferon alfa-2a in participants with chronic hepatitis B infection. J. Hepatol..

[139] Yuen M.F., Wong D.K., Schluep T., Lai C.L., Ferrari C., Locarnini S., Lo R.C., Gish R.G., Hamilton J., Wooddell C.I. (2021). Long-term serological, virological and histological responses to RNA inhibition by ARC-520 in Chinese chronic hepatitis B patients on entecavir treatment. Gut.

[140] Ganchua S.C., Paratala B., Iott C., Yuen M.F., Gane E., Eley T., Sims K., Gray K., Antoniello D., Lam A.M. (2022). Inhibition of hepatitis B surface antigen by RNA interference therapeutic AB-729 is associated with increased cytokine signatures in HBV DNA+ chronic hepatitis B patients. J. Hepatol..

[141] Ganchua S.C., Paratala B., Iott C., Gane E., Yuen M.F., Eley T., Sims K., Gray K., Antoniello D., Lam A.M. (2022). Reduction of hepatitis B surface antigen mediated by RNA interference therapeutic AB-729 in chronic hepatitis B patients is associated with T cell activation and a decline in exhausted CD8 T cells. J. Hepatol..

[142] Yuen M.F., Heo J., Kumada H., Suzuki F., Suzuki Y., Xie Q., Jia J., Karino Y., Hou J., Chayama K. (2022). Phase IIa, randomised, double-blind study of GSK3389404 in patients with chronic hepatitis B on stable nucleos(t)ide therapy. J. Hepatol..

[143] Gane E., Yuen M.F., Kim D.J., Chan H.L., Surujbally B., Pavlovic V., Das S., Triyatni M., Kazma R., Grippo J.F. (2021). Clinical Study of Single Stranded Oligonucleotide RO7062931 in Healthy Volunteers and Patients with Chronic Hepatitis B. Hepatology.

[144] Vaillant A. (2022). Targeting Subviral Particles: A Critical Step in Achieving HBV Functional Cure but Where Are We with Current Agents in Clinical Development?. Viruses.

[145] Yuen M.F., Heo J., Jang J.W., Yoon J.H., Kweon Y.O., Park S.J., Tami Y., You S., Yates P., Tao Y. (2021). Safety, tolerability and antiviral activity of the antisense oligonucleotide bepirovirsen in patients with chronic hepatitis B: A phase 2 randomized controlled trial. Nat. Med..

[146] Kastelein J.J., Wedel M.K., Baker B.F., Su J., Bradley J.D., Yu R.Z., Chuang E., Graham M.J., Crooke R.M. (2006). Potent reduction of apolipoprotein B and low-density lipoprotein cholesterol by short-term administration of an antisense inhibitor of apolipoprotein B. Circulation.

[147] Ackermann E.J., Guo S., Benson M.D., Booten S., Freier S., Hughes S.G., Kim T.W., Jesse Kwoh T., Matson J., Norris D. (2016). Suppressing transthyretin production in mice, monkeys and humans using 2nd-Generation antisense oligonucleotides. Amyloid.

[148] You S., Singh J., Smith S., Jordan W., Remingler K., Joshi S., Ermler M., Delahaye J., Taylor A., Chakraborty S. (2021). Treatment with GSK3228836 Leads to HBsAg Reduction and Induction of Interferon Gamma Related Proteins and Chemokines in a Phase 2a, Randomized, Double-Blind, Placebo-Controlled Study. J. Hepatol..

[149] Gane E.J., Kim H.J., Visvanathan K., Kim Y.J., Nguyen A.H., Wallin J.J., Chen D.Y., McDonald C., Arora P., Tan S.K. (2021). Safety, Pharmacokinetics, and Pharmacodynamics of the Oral TLR8 Agonist Selgantolimod in Chronic Hepatitis B. Hepatology.

[150] Ayithan N., Ghosh A., Dwivedi A., Wallin J.J., Tan S.K., Chen D., Kottilil S., Poonia B. (2021). Oral Selective TLR8 Agonist Selgantolimod Induces Multiple Immune Cell Responses in Humans. Viruses.

[151] Robbins M., Judge A., Ambegia E., Choi C., Yaworski E., Palmer L., McClintock K., MacLachlan I. (2008). Misinterpreting the therapeutic effects of small interfering RNA caused by immune stimulation. Hum. Gene Ther..

